# Low-protein diets for chronic kidney disease patients: the Italian experience

**DOI:** 10.1186/s12882-016-0280-0

**Published:** 2016-07-11

**Authors:** Vincenzo Bellizzi, Adamasco Cupisti, Francesco Locatelli, Piergiorgio Bolasco, Giuliano Brunori, Giovanni Cancarini, Stefania Caria, Luca De Nicola, Biagio R. Di Iorio, Lucia Di Micco, Enrico Fiaccadori, Giacomo Garibotto, Marcora Mandreoli, Roberto Minutolo, Lamberto Oldrizzi, Giorgina B. Piccoli, Giuseppe Quintaliani, Domenico Santoro, Serena Torraca, Battista F. Viola, on behalf of the “Conservative Treatment of CKD” study group of the Italian Society of Nephrology

**Affiliations:** Nephrology Unit, University Hospital “San Giovanni di Dio e Ruggi d’Aragona”, Via San Leonardo, 84131 Salerno, Italy; Department of Clinical and Experimental Medicine, University of Pisa, Pisa, Italy; Nephrology Department, Alessandro Manzoni Hospital, Lecco, Italy; Territorial Department of Nephrology and Dialysis, ASL Cagliari, Italy; Nephrology Unit, Santa Chiara Hospital, Trento, Italy; O.U. Nephrology, A.O. Spedali Civili Brescia and University of Brescia, Brescia, Italy; Nephrology Division, Second University of Naples, Naples, Italy; Nephrology Unit, Landolfi Hospital, Solofra (AV), Italy; Pathophysiology of Renal Failure Unit, University of Parma, Parma, Italy; Nephrology Unit, University of Genoa and IRCCS A.O.U. San Martino IST, Genoa, Italy; Nephrology and Dialysis Unit, Ospedale S. Maria della Scaletta, Imola (BO), Italy; O.U. Nephrology and Dialysis, Fracastoro Hospital, San Bonifacio (VR), Italy; Department of Clinical and Biological Sciences, University of Torino, Torino, Italy; Nephrologie, CH Le Mans, Le Mans France, Italy; O.U. Nephrology, Dialysis and Transplantation, Santa Maria della Misericordia Hospital, Udine, Italy; Perugia Department of Internal Medicine, University of Messina, Messina, Italy

## Abstract

**Background:**

Nutritional treatment has always represented a major feature of CKD management. Over the decades, the use of nutritional treatment in CKD patients has been marked by several goals. The first of these include the attainment of metabolic and fluid control together with the prevention and correction of signs, symptoms and complications of advanced CKD. The aim of this first stage is the prevention of malnutrition and a delay in the commencement of dialysis. Subsequently, nutritional manipulations have also been applied in association with other therapeutic interventions in an attempt to control several cardiovascular risk factors associated with CKD and to improve the patient's overall outcome. Over time and in reference to multiple aims, the modalities of nutritional treatment have been focused not only on protein intake but also on other nutrients.

**Discussion:**

This paper describes the pathophysiological basis and rationale of nutritional treatment in CKD and also provides a report on extensive experience in the field of renal diets in Italy, with special attention given to approaches in clinical practice and management.

**Summary:**

Italian nephrologists have a longstanding tradition in implementing low protein diets in the treatment of CKD patients, with the principle objective of alleviating uremic symptoms, improving nutritional status and also a possibility of slowing down the progression of CKD or delaying the start of dialysis. A renewed interest in this field is based on the aim of implementing a wider nutritional therapy other than only reducing the protein intake, paying careful attention to factors such as energy intake, the quality of proteins and phosphate and sodium intakes, making today’s low-protein diet program much more ambitious than previous. The motivation was the reduction in progression of renal insufficiency through reduction of proteinuria, a better control of blood pressure values and also through correction of metabolic acidosis. One major goal of the flexible and innovative Italian approach to the low-protein diet in CKD patients is the improvement of patient adherence, a crucial factor in the successful implementation of a low-protein diet program.

## Background

### Historical background of low-protein diets for renal diseases

Dietary protein restriction is the mainstay of the nutritional therapy for CKD. Since the 19^th^ century it had been realized that uremic syndrome derives from the retention of molecules and toxins resulting from the catabolism of exogenous proteins, usually excreted with the urine. However, it was only in the 1960s, that Giovannetti and Maggiore suggested the low-protein diet as a therapy for advanced CKD [1]. At that time dialysis was still in the experimental stage and only a small number of patients could benefit. A protein restricted diet providing adequate amounts of aminoacids and energy supply was therefore the only widespread means to alleviate uremic symptoms and to prolong survival [1].

Thereafter, dialysis techniques and facilities, as well as kidney transplantation, rapidly developed as replacement therapies for ESRD. Although these therapies came to represent milestones in the history of modern medicine, at the same time they reduced interest in the low-protein diet as a treatment for advanced CKD. Nonetheless, with the aid of the Brenner theory and its central aim of reducing protein-related glomerular hyperfiltration and hypertrophy, the low-protein diet was seen as a means to protect residual renal function and to slow down the CKD progression to ESRD [2]. Later, the availability of renoprotective and anti-proteinuric drugs (RAS inhibitors) again limited the implementation of dietary intervention in CKD. However, the low-protein diet still remains a viable means in the prevention or treatment of several metabolic and clinical abnormalities during CKD, as well as in the reduction of proteinuria, either in association with RAS inhibitors or in patients who cannot be treated with these drugs [3].

It is noteworthy that urea (as a marker of uremic toxins) reduction is not the only aim of the low protein diet. Indeed, in the early 1980s Maschio and Barsotti highlighted the importance of protein restriction in the reduction of phosphorus intake in moderate to advanced CKD [4, 5]. This aspect, neglected for many years, now enjoys significant renewed interest stimulated by the evidence of the key role of phosphorus retention in the pathogenesis of the so-called CKD-MBD and in the progression of renal disease [6, 7].

Dietary sodium restriction is another aspect of the nutritional therapy for CKD, as it allows better management of sodium and water retention, blood pressure control, and reduction of proteinuria [8].

Last but not least, it is of paramount importance to underline that the renal diet is not only a matter of restriction. It is also essential to ensure that the full energy requirement is met in CKD patients (whether on low-protein or following autonomous diets), in order to prevent protein catabolism, maintain neutral nitrogen balance, and to maintain adequate nutritional status and body composition [9]. Contrary to just prescribing a low-protein diet, a comprehensive nutritional approach for CKD patients is to be considered mandatory.

An important improvement in the dietary treatment for advanced CKD was the availability of protein-free foods [10]. These products consist of carbohydrates (starch) and are virtually free of nitrogen, potassium, phosphorus and are also low in salt content. They effectively replace analogous staple foods (bread, pasta, biscuits) making it possible to reduce the intake of protein of low biological value, and thereby allowing adequate intake of animal proteins whilst ensuring a high energy intake. Such protein-free products have been widely available in Italy for some time and, nowadays, can also be obtained in other countries around the world. In the early years, protein-free products were far less palatable than corresponding foods, thus limiting patient adherence. In the last few years, however, these foods were further developed and their palatability much improved. It is in fact notable that the risk of “malnutrition” associated with a low-protein diet is usually the result of an insufficient energy intake rather than of a low protein supply. Hence, when protein-free products are refused by patients or are unavailable, a vegan diet can be offered as an alternative, providing low protein (0.7/kg/dayay), low-phosphorus and an adequate support of essential aminoacids derived from a strict combination of cereals and legumes [11]. At the same total protein content, the vegan diet allows more favourable effects including a lesser net acid production, a greater anti-proteinuric effect and a lower net intestinal absorption of phosphorus [12]. The observation that the vegan diet has effects similar to that of a conventional low-protein diet also permits an alternation between the two, thereby increasing the choice of foods and, consequently, the patients’ adherence [13].

Finally, when a modest restriction of protein and phosphorus is not enough (as in the case of severe reduction of renal function), a very low-protein (0.3 g/kg/day), very low-phosphorus diet supplemented with calcium, folates, group B vitamins and mixtures of essential aminoacids and ketoacids, can be prescribed [14, 15].

Though low-protein diets supply low amounts of proteins they are high in carbohydrates, which are contraindicated for patients with nephrotic syndrome or diabetes mellitus. However, in the late 1980s the beneficial effects of protein restriction on glucose metabolism [16–18], and also proteinuria [19–22] were proven. Hence, the change in the quantity and quality of dietary proteins is indicated even in diabetic and proteinuric renal diseases.

In summary, protein restriction, including reduction of phosphorus and salt, together with adequacy of energy intake, represent the general characteristics of the nutritional therapy for CKD patients. Moreover, the severity of restrictions and the amount and quality of food and supplements should be defined in accordance with the advancement of CKD and the clinical conditions under which it is observed.

Epidemiological data show that CKD is very common, particularly in elderly patients with comorbidities, including cardiovascular diseases, diabetes and hypertension. At the present time, the CKD population is much older in comparison to previous decades [23, 24], a factor that introduces psychological and socio-economic elements that must be dealt with. Thus, the nutritional management of renal patients becomes more and more complicated and far removed from a simple schematic dietary plan solely related to the degree of renal impairment [14]. Nutritional treatment must be increasingly focused and adapted to patient characteristics as well as to clinical and extra-clinical needs. This is in order to obtain the maximum benefits whilst minimizing the risks, as well as to achieve satisfactory patient adherence to dietary prescriptions [25].

Although the effect on lowering the GFR decline rate seems small [26, 27], protein restriction appears capable of reducing by 31 % the relative risk of initiating dialysis [28]. In addition, in patients with ESRD, a low protein regimen can also allow a reduction in the frequency of dialysis [29–31]. Ultimately, these results are of great interest to the patient, nephrologist and health care system alike.

## Discussion

### Rationale and goals of nutritional treatment in chronic renal insufficiency

#### Protein waste products in the pathophysiology of renal failure

With the aim of understanding the possible role of diet in controlling uremic symptoms and reducing the rate of progression towards end stage renal disease, one should consider separately each dietary component. These are: protein waste products, phosphate, sodium, and acid load.

Uremic syndrome is due to progressive reduction in renal function, leading to impairment of several biochemical and physiological functions. Impaired kidney function reduces the excretion of many compounds that consequently accumulate in extra- and intracellular compartments and which contribute to the development of the uremic syndrome. These uremic toxins mainly include protein-bound substances and free water-soluble molecules of low to medium molecular weight, [32–34], most of which derive from protein breakdown [35]. Thus the reduction of protein intake can reduce either the accumulation of these toxins or the onset or severity of uremic symptoms and complications.

Another possible source of aminoacid/protein derived toxic compounds is the bacterial fermentation of proteins in the intestine, leading to increased indoxyl sulfate and p-cresyl sulfate serum levels [36, 37]. These toxins have been associated with CKD progression and CKD-related complications, and could contribute to an acceleration in cardiovascular disease and uremic bone disease. The predictive role of baseline indoxyl sulfate and p-cresyl sulfate levels for CKD progression have been found to be significant, even after adjusting for age, gender, diabetes status, albumin levels, eGFR, calcium x phosphorous product, PTH, hemoglobin, and high-sensitivity C-reactive protein [38]. Moreover, serum levels of indoxyl sulfate are associated with overall and cardiovascular mortality [39]. Reduced renal function determines the increase of circulating uremic toxins, which may promote the CKD progression (a self-reinforcing or vicious circle).

#### Phosphate in the pathophysiology of renal failure

Phosphate is mainly found in foods with high protein content, such as meat and cheese. The kidneys regulate serum phosphate concentration by modulating its urinary excretion and balancing the net phosphate absorption from the gastrointestinal tract. In the past, concerns regarding the consequences of phosphate retention in moderate to advanced CKD were mainly focused on its negative effect on bone mineralization. Excess of phosphate causes the increase of FGF-23, inhibits the production of 1,25(OH)D3, increases PTH, and alters polyamine metabolism [40]. In previous years greater attention was paid to the cardiovascular effect of impaired mineral metabolism and a comprehensive nomenclature, CKD-MBD, was given to this complication [41]. It has been accepted that the major role of phosphate retention on patient outcome is due to its unfavorable effects on arterial and coronary calcification, left ventricular mass, cardiac fibrosis and possibly CKD progression [42, 43].

#### Sodium in the pathophysiology of renal failure

A reduced kidney function increases the body total sodium pool and causes hypertension and edema; this is in part due to reduction in GFR and in part to the activation of the sympathetic nervous system and the renin-angiotensin-aldosterone system. A high salt intake increases the amount of urinary protein excretion [44, 45], a major risk factor for the onset and progression of both kidney and cardiovascular disease. A double blind study demonstrated that by reducing salt intake from 10 to 5 g/day urine protein excretion reduces by about 20 %. A subsequent double blind study showed that an even smaller reduction in salt intake, from 9.7 to 6.5 g/day, reduces urinary albumin excretion in people with mild hypertension [46, 47].

#### Metabolic acidosis in the pathophysiology of renal failure

Acid excretion (mainly as phosphates and sulphates) and the regeneration of bicarbonate both decrease with lowering renal function, leading to metabolic acidosis that has several negative consequences, including bone mineral loss, muscle wasting, reduced protein anabolism and increased protein catabolism. All these changes contribute to protein malnutrition and progressive impairment of nutritional status. Another significant effect of acidosis is the shift of potassium from the intracellular to the interstitial fluid, causing hyperkalemia, and hyperphosphatemia. Finally, evidence exists that metabolic acidosis also contributes to the progression of renal damage [48–50].

#### Protein and aminoacids metabolism in chronic renal insufficiency

Humans are able to maintain lean body mass and body protein balance over a broad range of dietary protein intakes. Studies performed by whole body, steady-state, stable isotope infusions have shown that the human body responds to a reduced protein intake with integrated and adaptive metabolic changes. These include a reduction in aminoacid oxidation with more efficient use of aminoacids derived from protein degradation, a decrease in protein degradation and, ultimately, a decrease in whole body protein synthesis [51, 52]. Impaired ability to activate these adaptive mechanisms in patients with renal function impairment would compromise nitrogen preservation when nutrient/protein intake is reduced. The understanding of how renal function impairment affects the body’s ability to respond to changes in nutrient intake is relevant since low-protein, high-calorie diets are commonly used in conservative CKD treatment.

Nutritional impairments, such as malnutrition or cachexia, are more often clinically evident in advanced CKD (stage 4–5) and even more at the beginning of renal replacement treatment. Mechanisms causing muscle protein loss are complex and not always associated with anorexia, but they are linked to several abnormalities that stimulate protein degradation and/or decrease of protein synthesis [53]. During the CKD course, the loss of kidney excretory and metabolic functions is associated with the activation of some pathways of endothelial damage, including inflammation, acidosis, resistance to anabolic hormones, and defective insulin signalling [54]. These factors may overlap those already operating in ageing and in comorbid conditions, such as diabetes, inflammation and infections, favouring protein energy wasting [55, 56]. Recent advances in experimental uraemia have consistently increased our understanding on intracellular pathways producing loss of muscle mass. Metabolic acidosis increases aminoacid oxidation and protein degradation through an upregulation of the ATP-dependent, ubiquitin-requiring pathway [53]. In addition, impaired signalling through the insulin receptor substrate-1/phosphoinositide 3-kinase/Akt pathway can predispose to catabolism through the up-regulation of atrogin-related proteolytic pathways [57]. These effects may be especially injurious in uremia, where impaired IGF-I signalling decreases the proliferation of satellite cells and blunts muscle remodelling and regeneration [58, 59].

Uremia-specific alterations in muscle aminoacid metabolism can also accelerate catabolic processes. Low levels of ketoisocaproate, ketoisovalerate and ketomethylvalerate, the respective ketoanalogs of leucine, valine and isoleucine, all occur in plasma and in muscle cells of uremic patients [60]. A reduced release of valine and leucine and their ketoacids from muscle is responsible for their reduced blood levels [60]. These abnormalities arise largely because of impaired dietary intake, urinary protein losses, reduced renal metabolism and synthesis, and increased BCAA catabolism in muscle [53].

Experimental evidence shows that, provided metabolic acidosis is corrected and energy intake is adequate, patients with advanced stage CKD can fully adapt to low protein regimens. Several nitrogen balance studies have shown that patients with advanced CKD can maintain neutral or slightly positive N balance with protein intakes as low as 0.55-0.6 g/Kg [52]. Indirect data suggest that the mechanisms by which protein turnover adapts to a low protein intake can be impaired in the presence of metabolic acidosis [61]. Other studies indicate that when acidosis is corrected, the adaptation of protein metabolism to low protein diets is unimpaired. Indeed, in non-acidotic patients with moderate renal impairment the adaptive response to a standard low-protein diet (0.6 g/kg/day) include a normal decline both in the rates of whole body leucine oxidation and protein degradation [62, 63].

Two studies indicate that protein metabolism and N balance adapt successfully also in patients with uncomplicated CKD who are compliant to a VLPD supplemented with essential aminoacids and ketoacids. Masud et al. administered a VLPD (0.28 g/protein/kg) supplemented with ketoacids or aminoacids in non-acidotic CKD patients [64]. When patients were studied 3 weeks after the start of VLPD regimen, they found that body nitrogen balance was neutral and that this was achieved by a marked reduction in whole body aminoacid oxidation and postprandial inhibition of protein degradation. In a second study, the long-term adaptive response of six patients to a VLPD supplemented diet was evaluated [9]. After more than one year of very low protein diet, the rate of body N balance was neutral and did not differ from basal values. In addition, the whole protein synthesis, protein degradation and leucine oxidation were similar to baseline. Also, in the long-term, the neutral N balance was maintained by a suppression of aminoacid oxidation and inhibition of protein degradation. As a whole, the evidence existing in the literature shows that low-protein diets (up to 0.3 g protein/kg) are nutritionally safe during advanced CKD provided there is sufficient energy and aminoacid intake and correction of metabolic acidosis.

#### Adaptation to low protein diets: energy metabolism in chronic renal insufficiency

Energy metabolism in CKD is to some extent still unclear. Resting energy expenditure in stable patients with chronic renal failure on conservative treatment does not differ from control subjects [65]. However, patients with advanced CKD may develop protein-energy wasting and low dietary energy and nutrient intakes are often associated. It is possible that low energy intake represents an adaptive response to reduced energy needs. Alternatively, EE could be normal or high and a low energy intake would be inappropriate. Studies aimed at evaluating EE measurement in renal patients in the resting state, as well as during mild to vigorous exercise, confirm that no difference exists when EE in patients with advanced renal insufficiency is compared to that of normal subjects. These findings suggest that for a given physical activity, energy expenditure in non-dialyzed, chronically uremic patients should not be different from normal [65]. Therefore, the low energy intake of many of these patients may be inadequate for their needs, causing a negative energy balance that in turn leads to protein-energy wasting [66].

The association between dietary energy and protein metabolism was evaluated in clinically stable, non-dialyzed CKD patients fed with different dietary energy intakes (45, 35, 25 or 15 kcal/kg/day), at the same low protein intake (0.55 to 0.60 g/kg/day [67]. Nitrogen balance correlated directly with energy intake, and although some clinically stable non-dialyzed chronic uremic patients ingesting 0.55 to 0.60 g protein/kg/day may maintain a neutral nitrogen balance with an energy intake below 30 kcal/kg/day, a dietary intake providing approximately 35 kcal/kg/day is more likely to maintain neutral or positive nitrogen balance, maintain or increase body mass, and reduce net urea generation. Thus, a crucial aspect of nutrient intake in CKD is the amount of energy supply. Essentially, in the presence of a reduced protein intake, a neutral or positive nitrogen balance requires high energy intake, whereas a low energy intake causes protein degradation, negative nitrogen balance and loss of lean body mass.

There is evidence that many patients with CKD spontaneously reduce their energy intake as GFR decreases [68]. The failure to maintain adequate energy intake may be due to several factors including: anorexia, nausea, fatigue, anemia, restrictive therapeutic diets, co-morbid diseases and psychosocial factors. The age of CKD patients makes the energy intake pivotal in maintaining an acceptable nutritional status. Indeed, being elderly induces a worsening in dietary habits because of several factors, such as depression, anorexia, delayed gastric emptying, altered gastric distension, hormonal changes, taste and smell deterioration, pill burden and insulin resistance. Consequently, elderly patients with advanced renal insufficiency are at very high risk of protein energy wasting. Besides age, also of relevance in the prescription of energy intake is the strict relation between EE, gender and physical activity of which must also be taken in consideration. Indeed, daily EE is lower by 8-14 % and 16-20 % in sedentary adult women and men respectively. In addition, elderly people show a decline in resting EE (linked to a 3 % loss of lean body mass per decade) [69]. Based on this evidence, the most recent dietary reference intakes (Food & Nutrition board) provide the mean individual energy requirement for a 30-year old adult, from which the requirements for older women and men can be calculated by reducing the value for 30-year olds by 7 and 10 kcal per additional year of age, respectively [70].

Finally, there is a metabolic adaptation to reduced energy intake, which includes a decrease in resting EE due to the loss of lean body mass and to an improved efficiency of energy metabolism. Thus, when the energy intake does not reach the recommended values, malnourished CKD patients benefit from any increase in their nutritional intake, mainly a high energy supply.

As to the role of LPD, it has been shown that LPD ncreases insulin-mediated glucose disposal in CKD [71]. LPD ameliorates insulin sensitivity in renal insufficiency, even for low plasma insulin concentrations. The causative role of LPD-induced metabolic changes is not yet well known. Nonetheless, since the rate of energy production is increased by LPD, the energy intake should be increased when protein intake is restricted.

In summary, full compliance with patient energy requirement is mandatory for correct adaptation of protein metabolism to dietary protein restriction. The recommended daily energy intake in CKD should be 35 kcal/kg/day for patients aged < 60 years; and 30 kcal/kg/day for those aged > 60 years. These recommendations for energy intake must be adapted to the individual patient’s needs, and will mostly depend on the level of physical activity.

### Strategies for different low-protein diets for CKD in Italy (Table 1)

**Table 1:** Dietary composition of low-protein diets for CKD patients

	Normal diet	LPD	Vegan	VLPD
Nutrients				
Energy requirement	normal	high	high	high
Protein, g/Kg/d	0.8	0.6	0.7	0.3-0.4
Prevalent origin of proteins	Mixed	Animal	Plant	Plant
Phosphate, mg/d	700-800	500-600	500-600	300-400
Sodium, mmol/d	100	100	100	100
Supplements				
Free-protein products use	Optional	Yes	No	Yes
EAA + KA	No	Optional	Optional	Yes
Calcium, g/d	Optional	0.5-1.0	0.5-1.0	0.5-1.0
B12 Vitamin	No	Optional	Yes	Yes
Iron	No	Optional	Yes	Yes

*EAA* essential aminoacids, *KA* ketoacids

#### Normal-protein, normal-sodium diet

In healthy adults the RDA for proteins has been set at 0.8 g/kg/day, which meets metabolic requirements in 97.5 % of the adult population [72]. Recommendations on dietary salt intake for the general population have also been recently issued, mainly on the basis of cardiovascular effects associated with different levels of salt intake [73]. Generally, the recommendation is for a reduction in sodium intake to < 100 mmoL/d (i.e., < 5.8 g/day sodium chloride). Since preliminary evidence seems to indicate that severe reductions (<6 g/day) may not be safe [74], optimal dietary salt should be 4–6 g/day. Therefore, a “normal” diet for healthy adults in the general population should contain 0.8 g/kg/day proteins and nearly 6 g/day of salt.

Practical fulfilment of these recommendations does however prove to be far from theory. Typical PI in developed countries are as much as 3.0 g/kg/day [75]. In Italy, a small study showed a mean PI of 1.3 ± 0.6 g/kg/day, with 73 % and 26 % of subjects evidencing levels higher than 0.8 and 1.6 g/kg/day, respectively [76]. The picture for sodium intake is even worse. Indeed, analysis of surveys on sodium intake from 66 countries throughout the world has revealed that the estimated mean level of sodium consumption in 2010 was almost double the RDA value and accounted for nearly 10 % of deaths [77]. In the Italian adult population, average salt intake is 10.9 g/day in men and 8.5 g/day in women, with intakes > 5 g/day in 97 % and 87 %, respectively [78].

This complex scenario has led the new guidelines on CKD management to suggest an appropriate nutritional education program aimed at achieving a protein intake of 0.8 g/kg/day in all adults with GFR less than 30 ml/min/1.73 m^2^, whilst also avoiding a high protein intake (>1.3 g/kg/day) in all patients at risk of CKD progression [79]. Recommendations for CKD patients with higher GFR and/or at low risk of CKD progression remain undefined. When considering that most CKD patients have salt-sensitive hypertension [80], the only recommendation is a lowering of salt intake to 4–6 g/day, unless contraindicated (i.e. salt losers, volume depletion) [79].

However, guidelines are essentially based on findings from randomized trials, and “optimal” testing of the efficacy of nutritional interventions is difficult not only due to economic restrictions (limited or any funding for RCT on non-pharmacologic interventions) but also to problems inherent to the study (such as poor adherence to diet that ultimately affect results). With such ambiguity in efficacy it may be wise to resort to pathophysiology, which has demonstrated the importance of adapting dietary intakes to impaired renal function [81], and to “pragmatically” pursue in CKD the dietary goals established for the general population. Therefore, prescribing a “normal” diet should be the basic approach in most CKD patients. It is noteworthy that in patients with CKD and eGFR < 60 ml/min*1.73 m^2^, only one out of two complies with the 0.8 g/kg/day protein regimen and one out of five adheres to the 6 g/day dietary salt prescription despite regular nephrology care [82, 83].

Nevertheless, these findings should not prevent efforts in dietary intervention among nephrologists. Indeed, even small reductions in protein intake, such as a decrease of 0.2 g/kg/day versus usual diet, still allows significant improvements in metabolic profile and in the risk of renal death [3, 28]. Similarly, CKD patients under renin-angiotensin system inhibitors show better renal prognosis when adhering to salt restriction even if of moderate degree (7 to 10 g/day versus >12-14 g/day) [84, 85]. This holds true mainly because salt restriction potentiates the anti-proteinuric efficacy of RAS inhibitors [86].

Overall, these data and the certainty that effectiveness of nutritional therapy is impaired if most patients are not adherent, should stimulate nephrologists into prescribing the “normal” diet to all their CKD patients as the first approach, being aware that even small decreases in the protein-salt consumption are beneficial. Adherence to “normal” diet may guide future decisions on switching the patient to a more restricted, and potentially more effective, dietary regimen.

A “normal diet”, aiming at intakes of 0.8 g/kg/day of protein, normal energy intake, and 100 mmol/day of salt, can be easily prescribed to all CKD patients with the suggestion of avoiding “excessive” intakes of proteins [87]. Patient attention should also be drawn to the importance of healthy diets, namely a “Mediterranean” or “DASH” diet.

#### Conventional low-protein, low-phosphorus diet

The progressive loss of renal function during the course of CKD leads to defective excretion capacity of several waste products and minerals which accumulate in the body. When renal function in impaired, the renal handling of nutrient waste products and electrolytes is various. For instance, urea and organic acids accumulate as the GFR declines. In contrast, for other solutes such as phosphate and sodium, a body neutral balance is maintained (till advanced or even very advanced renal function decline) thanks to the activation of some pathophysiological, adaptive mechanisms that, however, are detrimental (“*trade-off hypothesis*”) for the kidney and the whole body (i.e. FGF-23 increase, secondary hyper-parathyroidism; extra-cellular volume expansion and consequent heart failure and arterial hypertension).

Major metabolic derangements in CKD occur from stage 3b. Most patients show overt secondary hyper-parathyroidism, increased potassium and phosphate serum levels, metabolic acidosis, anemia and sub-clinical water retention [88, 89]. In this phase of renal disease a basic renal diet (low-normal protein diet; see above) is no longer capable of neutralizing the increased retention of nutrient waste products and related clinical complications. Thus, in CKD 3b it is conceivable that a more restricted nutritional treatment can be started, with the purpose of reducing the load of those products which have impaired renal handling and thereby improving uremic signs and symptoms, uremic toxicity, preventing the onset of malnutrition, and, possibly, preventing or reducing the CV risk factors whilst also delaying the start of renal replacement treatment [14].

A “conventional low-protein diet” for adult patients with established CKD should contain: 0.6-0.7 g/kg/day of protein (>50 % at high biological value such as meat, fish and egg). To avoid a negative nitrogen balance with such a reduced protein intake, the energy intake should be in the normal-high range, that is, 30–35 kcal/kg/day, derived from lipids (around 35 % of the total amount preferably from vegetarian origin, with cholesterol less than 300 mg/day and saturated fat less than 5 %) and carbohydrates (around 55 %). This “diet” is also low in phosphate (600–800 mg/day), sodium (2–3 g/day), organic acids, potassium and calcium content and may, therefore, be supplemented with calcium (400–800 mg elemental calcium per day). To accomplish the goals of low protein, normal-high energy intake and balanced intakes of macronutrients at one and the same time, such a “conventional low-protein diet” requires the use of protein-free products for the main source of energy. Today these products are usually available as pasta, bread and flour but also as precooked soups and desserts and represent a very valuable resource for optimal low-protein dietary management of CKD, allowing high energy intake with no phosphate, absence of protein of low biological value, and a lower sodium burden.

The main role of a “conventional low-protein diet” in the moderate to advanced stages of CKD is based on its effect on the metabolic abnormalities of CKD. Even a slight reduction of protein intake of 0.2 g/kg/day achieves significant metabolic improvements on uremic state, metabolic acidosis and hyperphosphatemia [3]. Indeed, the protein prescription of 0.6 g/kg/day compared with a 0.8 g/kg/day protein diet allows a better control of hyperparathyroidism and metabolic acidosis, even if adherence to the diet is incomplete [90]. Also, a robust interaction has been demonstrated between nutrition and cardio-renal protection. In fact, in non-diabetic CKD patients receiving ACE*i*, the reduction of salt intake (to 6 g/day) enhances the urinary protein-lowering effect of ACE*i*, either alone or in dual angiotensin block [85]. Moreover, in patients from the REIN study, the urinary protein-lowering effect of ACE*i* declines as serum phosphorus increases, suggesting that phosphorus impacts the renoprotective effect of ACE*i* [7]. Hence, in CKD comprehensive nutritional treatment reduces uremic signs and symptoms and also allows a lessening of the CV risk.

Major concerns for this dietary regimen are adherence and safety. Patient compliance to a low-protein diet is low. Since major determinants of adherence are understanding (knowledge) and satisfaction of the patient (achieving their objective, receiving required support) [91], to pursue this aim requires skilled dietitians, individualized dietary programs, intensive educational programs and regular counselling [92], all of which require dedicated personnel and extra resources of time and funding [93] and are rarely available in common clinical practice [94]. An option for overcoming this limitation is found in simplified approaches to “conventional low-protein diet” which may be practiced when dietitians are not available [87, 95].

As for nutritional safety, given an adequate energy intake (i.e. in the normal-high range, 30–35 kcal/kg/day) and correct nutritional monitoring, it has been definitively demonstrated that malnutrition is extremely rare in CKD patients on a low-protein diet. In a randomized trial in which more than 400 CKD patients on a low-protein diet were followed for 30 months, only three subjects developed malnutrition (less than 1 %) [96]. Overall, the side effects of a “conventional low-protein diet” in CKD are negligible and can be easily prevented with careful, standard clinical monitoring (see above).

#### Vegan low-protein, low-phosphorus diet

As specified above, a conventional low-protein diet requires the use of protein-free products as the main energy source. Unfortunately in some circumstances these artificial foods may have poor palatability and be expensive and psychologically unacceptable to the patient, especially when consumed away from home . When the intake of protein-free products is inadequate, the unwanted consequence is low energy intake leading to loss of fat and lean body mass.

To overcome the need of protein-free foods, we implemented a vegetable-based, low-protein (0.7 g/kg body weight/day), high energy (30 to 35 kcal/kg/day) vegan diet. The major limitation of a vegetarian low-protein diet is the adequacy of essential aminoacid supply, since plant proteins are not considered as high biological-value proteins. This diet used a cereal-legume combination to meet the essential aminoacid requirement [11]. Such a combination may appear at first sight to be inadequate, as it is well known cereals are low-biological value proteins because they are poor in lysine content; and legumes are lacking in methionine. However, cereals are rich in methionine whereas legumes are rich in lysine, making these foods complementary in aminoacids composition. A precise combination of each, therefore, is fully capable of providing the essential aminoacids requirement.

Notably, at the same protein intake, plant origin proteins have a lower impact on glomerular permeability and hemodynamics than animal protein [97], and the net acid production is lower. A vegan diet has a favorable lipids and fatty acids composition and also contributes to the lowering blood pressure. Phosphate is mostly present as phytate, with a quite lower intestinal absorption rate, so reducing, even at equal phosphate content, the actual dietary phosphate load [12]. Of note, in this diet, meat or fish are not allowed, making iron and vitamin B12 supplements necessary.

The limitation of food choices in the vegan diet, as well as in the conventional low-protein diet, may prove too unimaginative and repetitive in the long-term and as such represents the main factor limiting patient adherence. Since the vegan diet achieved comparable results to the conventional low-protein diet, one suggestion is to alternate these two diet regimens. This strategy avoids the exclusion of a food category (namely cereals during conventional low-protein diet, or animal proteins during vegan diet), and reduces the need of protein-free foods [13]. On the whole, alternating vegan and conventional low-protein diets was well accepted by patients and it may represent a further practical alternative for many patients on long-term dietary protein restriction.

#### Very low-protein, very low-phosphorus supplemented diet

The VLPD is a vegetarian diet that includes protein-free artificial foods, vegetables, fruits and supplementation of essential aminoacids (EAA) and ketoacids [11]. The VLPD composition for an adult man of 70 kg body weight ensures 2100–2500 kcal, 21 g of proteins, 73-80 g of lipids (4 % saturated), 310-350 g of carbohydrates (12 % simple carbohydrates), 16 g of fibers, 30–40 mmol of sodium, 35–50 mmol of potassium, 350-450 mg of phosphate, 3-5 mg of iron and 200-250 mg of calcium. Supplements are administered as tablets containing L-lysine (105 mg), L-threonine (53 mg), L-histidine (38 mg), L-tyrosine (30 mg), hydroxy-methionine (59 mg), calcium-keto-valine (86 mg), calcium-keto-phenylalanine (68 mg), calcium-keto-leucine (101 mg), calcium-keto-isoleucine (101 mg). This formulation is the Alfa-Kappa® used in Italy, whereas globally Ketosteril® is used. The latter differs from the former by the presence of Tryptophan. In order to maintain a neutral nitrogen balance even at a very-low protein intake, tablets are given at the dose of 1 pill every 5 kg/bw [98]. Use of supplements allows nitrogen recycling as a means of building the essential aminoacids from the relative ketoacids, thus further reducing serum urea levels. Calcium content is 45 mg per tablet and the total calcium intake is about 630 mg in an adult man of 70 kg (75-80 % of RDA). Total nitrogen content is 37 mg. Due to the very low nitrogen intake, a high energy intake (never lower than 30–35 kcal/kg/day) is mandatory. Protein-free foods allow an energy supply of 30–35 kcal/kg of body weight with a low phosphate intake. Salty food, food with additives, game, giblets, chocolate, puddings, creams, sweets, liquors, dried and syrup fruit, bananas, figs, tangerines, pomegranates, olives, mushrooms, spinach and truffle are not allowed. Still water is suggested, while a half glass of red wine is permitted at lunch and dinner. A free-choice meal is allowed only once a week. Patients may also dine out, but they are required to ask the chef to refrain from adding salt, and are also expected to avoid sauces (such as ketchup, mustard, and mayonnaise), and to request that dishes are cooked in a simple way (grilled, roasted or boiled). To improve the taste of food it is permissible to use vinaigrette, celery, basil, parsley, marjoram, oregano, salvia, rosemary, garlic and onion.

VLPD showed several additional positive effects. There were lower indoxyl sulphate levels compared to usual LPD [99]; a decreased need for erythropoietin [100]; better control of blood pressure, possibly through a reduced salt intake [101]; lower urinary and plasma phosphate and FGF23 levels [102]; and lower proteinuria and better antiproteinuric response in patients assuming ACE*i* and sartans [103]. Moreover, vegetables and fruits increase plasma bicarbonate levels, thus improving the metabolic acidosis [104]. Interestingly, vegan-vegetarian supplemented LPD in pregnant women with CKD stages 3–5 reduced the risk of low gestational age babies and may counterbalance the worsening of proteinuria and kidney function associated with pregnancy related hyperfiltration [105]. Also, in diabetic patients vegan supplemented LPD facilitated better management of CKD progression whilst awaiting a combined pancreas-kidney transplant [106]. Overall, the supplemented VLPD has been demonstrated as safe during either the pre-dialysis CKD period or during the following long-term dialysis period for patients who previously received such a diet [107].

The VLPD has recently received a final consensus from experts in this field [14]. Obviously, VLPD management requires significant expertise by nephrologists, together with high patient compliance and acceptance of lifestyle changes, social and family support and, above all, a high doctor-patient empathy.

#### Pragmatic personalized low-protein dietary approaches

The input for re-designing moderately restricted, simplified low-protein diets (to only later switch to a severely restricted simplified low-protein diet) for CKD patients, came from either clinical factors (difficulty in attaining good dietary compliance) or resource-related issues (lack of dedicated dietitian).

The tenets forming the basis of the simplified diets were the following: to improve compliance, the diet should be qualitative and not quantitative (nothing needs to be weighed); the control of quantity of calories, protein, sodium and phosphate content is based upon clinical assessment, body weight control, diet diary, serum and 24 h urine parameter levels. One to three unrestricted meals per week are allowed, to ease the psychological burden of dietary restrictions and as a tool to limit the risk of under-nutrition. Indeed, the example of the Okinawa diet suggests that occasional intake of particular nutrients may be favourable, thus overcoming the concept of daily needs [108]. The choice of the type of diet depends upon the patient’s preferences. A moderately restricted diet may be the first step towards a further reduction (i.e. a very low-protein diet), which combines a vegan approach and protein-free commercial food. The addition of the combined ketoacids and aminoacids supplements allows for erring “*on the safe side*” as regards integration of different plant-derived proteins, thus permitting a purely qualitative approach in relation to forbidden and allowed food. Such a qualitative approach is also used for diets based upon the substitution of pasta and bread with protein-free food. Other diets, whether vegan, traditional or tailor-made require a qualitative and quantitative approach.

Starting from situations in which conventional low-protein diets were rarely employed, such as with type 1 diabetic patients and pregnant patients [106, 109], a multiple choice, stepwise treatment approach has been developed. While diet composition is discussed elsewhere in detail [87], four major points may summarize this approach [110, 111]. The strategy is centred on the low-protein diets considered on the whole as an integrated menu in which patients should chose the “diet” which best suited their preferences, according to the following suggestions: 1) chose the diet you prefer (or that which bothers you least); 2) change it if you want to try a different approach; 3) do not weigh food (qualitative approach); 4) enjoy 1–3 unrestricted meals per week. To facilitate the shift among different diets, these are built with a common frame plus a variable part which satisfies the different protein targets (0.6 with protein-free food; 0.6 vegan supplemented; 0.6-0.8 vegan non supplemented; 0.3 vegan with protein-free products supplemented with essential amino acid and ketoacids). Patients chose different diets when these options of protein restriction were offered. In agreement with experience gathered from other vegan diets and with alternation of vegan diets, and of diets using commercial protein-free food, this approach provides for good compliance (protein intake aimed at 0.6 g/kg/day of proteins; estimated as 0.5-0.7 g/kg/day), possibly because the multiple choice and the variety of different diets empowers the patients [11, 13, 105, 111]. In a recent report on over 800 patient-years of observation where such a multiple choice, stepwise dietary approach was applied, about 50 % of the patients were still on follow-up after the first finding of eGFR below 15 mL/min, previously considered as the marker of “early” dialysis initiation, and about 25 % were still on the diet for four or more years (50 % at 1 and 25 % at 2 years considering eGFR below 10 mL/min) [112].

### Dietary approach in special groups of CKD patients

#### Diabetes

Diet and lifestyle modifications are key components of DM care and an optimal nutritional approach helps the management of blood glucose, lipids, body weight and blood pressure, thus reducing both the incidence and progression of DM complications, including CKD.

The Italian Guidelines for DM Care suggest that people at high risk of diabetes should be encouraged to follow a diet rich in fiber from vegetables, fruits and whole grains and low in animal fats. The first step in managing a diet for a DM patient in an early stage of CKD is to ensure that carbohydrate intake is within the recommended range of 45 %-60 % of total calories and the actual fiber intake is similar to that of the general population (14 g/1000 kcal/day) [113]. Dietary sodium and protein restriction should be a mainstay in the nutrition therapy of all patients with proteinuria and it has proven effective even in overt diabetic nephropathy [85, 114–116]. When CKD progresses, a high protein intake is detrimental for the progressive decline of renal function. Indeed, the Italian DM Standard Care [113], and the Canadian Diabetes Association guidelines [117], recommend a LPD in the clinical management of DM patients with CKD although the benefits of protein-restricted diets have long been debated [118, 119].

A recent Italian study in 74 older adults with T2DM and CKD stages 3b-4 demonstrated the feasibility and the adherence to a moderate protein diet (0,7 g/kg/day for only 6 days/week); LPD regimen significantly reduced renal failure by 42 % [116]. This beneficial effect is also associated with an improvement in low-grade inflammation, oxidative stress, and proteinuria, without worsening glycemic control or nutritional status, demonstrating its sustainability in the long term [116]. The Italian clinical experiences started in the 1980s with the use of a VLPD, low phosphorus and supplemented diet, and involved both type 1 and type 2 DM patients [16, 120]. It was reported that a VLPD (protein 0.3 g/kg/day; phosphorus 5 mg/kg/day), supplemented with essential aminoacids and ketoacids, reduced the decline of creatinine clearance in eight self-controlled T1DM patients with renal failure due to overt diabetic nephropathy [16]. Later, in 22 T1DM and 10 T2DM patients, a beneficial effect on the progression rate of renal failure and proteinuria was proven for a protein restricted diet mostly consisting of foods of vegetable origin, [120]. The Authors tested two different patterns of diet (scheme A: 0.3 g protein/kg/day; phosphorus 5.5-6 mg/kg/day; scheme B: 0.7 g protein/kg/day; phosphorus 8.5-10 mg/kg/day). The B diet was a pure vegetarian diet with complementary proteins (from cereals and legumes) which meets completely the recommended daily intake of essential aminoacids. The Authors suggest that a moderately low protein vegetarian diet, such as diet B, might represent the first choice in the early stages of CKD due to diabetic nephropathy, while diet A is to be preferred in patients with a GFR <20 mL/min for delaying dialysis initiation. Overall, a recent meta-analysis on 13 RCT and 779 patients affected by diabetes mellitus indicates that the low-protein diet in such patients is significantly associated with improvement of diabetic nephropathy and moreover, is safe [115].

In conclusion, the dietary management of diabetic patients with CKD includes limitation of sodium intake to 1,5-2 g/day; an amount of carbohydrates equal to 45-60 % of total energy supply; reduction of saturated and trans fatty acids; with a fibre intake of 14-25 g/day being highly recommended. Daily protein intake should be approximately 0.8 g/kg/day in CKD stages 1–2 (lower normal intake) and reduced to 0.7- 0.6 g/kg/day in CKD stages 3–4; phosphorus content should be reduced to < 800-1000 mg/day. In selected CKD patients with CKD stage 5 and who were not in dialysis, a VLPD diet supplemented with aminoacids and ketoacids under strict clinical and nutritional follow-up, is a viable option.

#### Nephrotic syndrome

Edema is a major clinical manifestation of the nephrotic syndrome, due to extracellular fluid expansion secondary to sodium and water retention. For this reason dietary sodium restriction (2 to 3 g/day) should be recommended. In the DASH diet the limitation of red meat consumption is a strategy for reducing not only total and saturated fat intakes but also sodium intake, especially from processed meats [121]. Moreover, the DASH diet emphasizes increased consumption of protein from vegetables, poultry and fish wherein the quantity of sodium is lower [122].

In nephrotic syndrome, dietary intervention should also include a modified quantity and quality of protein intake through LPD. In some experimental models, LPD showed effective in decreasing the urinary protein excretion [123, 124], and this effect of LPD is synergic with ARBs agonist effects [125]. Human clinical trials proposed the LPD in proteinuric patients due to its effect on albumin homeostasis. Indeed, it was shown that in nephrotic patients the LPD results in decreased urinary albumin excretion in excess of any reduction in creatinine clearance and increase in plasma albumin mass [19]. In nephrotic syndrome, a reduced protein intake is also beneficial because of the increased synthesis of albumin, reduced proteolysis and the resulting increase in serum albumin levels [126, 127].

As well as in the experimental studies, the contemporary use of ACE*i* and LPD seems to have an additive beneficial effect on proteinuria. Indeed, LPD induces afferent arteriole vasoconstriction whilst ACE*i* induce post-glomerular vasodilatation [128]. Also, sodium restriction has an anti-proteinuric effect, either alone by itself or additive to that of ACE*i* and ARBs. To be sure, sodium restriction plus an inhibitor of RAS equalled (or even overcame) the effect of a dual blockade of renin-angiotensin-system on proteinuria, at the same time also limiting the risk of hyperkalemia. The reduction in proteinuria due to both sodium and protein restriction occurs to the same degree also in CKD diabetic patients and seems to be independent from changes in blood pressure [129, 130]. Also the VLPD supplemented with ketoacids is effective in reducing nephrotic proteinuria; the saving in terms of reduced protein excretion results in a delay of commencement of dialysis independent of the use of ACE*i* or ARBs [131, 132].

A vegan low-protein (0.7 g/kg/day), low-sodium diet for nephrotic patients even with preserved renal function, has also been proposed [20]. This diet was supplemented with essential aminoacids and ketoacids to partially cover the protein losses, so protecting against nutritional impairment. Urine protein losses decreased as well as LDL cholesterol levels, without detrimental effect on serum albumin. Finally, a non-supplemented vegan diet using soy as main protein source, exerted similar favourable anti-proteinuric and lipid-lowering effects [22].

Therefore, in proteinuric patients with normal or impaired renal function, it is reasonable to suggest diverse low-protein diets, integrating the urine protein losses and monitoring the nutritional status. In addition, the use of ACE*i* and LPD together seems to have an add-on beneficial effect on proteinuria, but with different mechanisms [128, 132].

In conclusion, sodium restriction represents the cornerstone of dietary approach in nephrotic patients, and changes in quality and quantity of dietary proteins may be an additional, effective intervention associated with the use of ACE*i* and/or ARBs.

#### ESRD: mixed dietary-dialysis approach

Giovannetti et al. and Locatelli et al. tested ESRD patients for a combination of dietary treatment plus a once-weekly hemodialysis schedule [29, 30]. The rationale was to ensure adequate metabolic control by combining a toxin load reduction “upstream” with a “valley” purification [133], while preserving as much as possible the residual renal function through avoiding relevant ultrafiltration rates during the dialysis session. This strategy can be considered as a forerunner of an “incremental” dialysis program.

The integrated Dialysis-Diet program consisted in a very low-protein diet, 0.3-0.4 g/kg/day, supplemented with essential aminoacids and ketoacids for 6 days a week, severe sodium and water restriction, and a once-a-week hemodialysis session [29, 30]. The program included patients with very-low residual renal function, below 5 ml/min. An unrestricted protein intake was permitted and mandatory on the day of dialysis treatment. This free-diet day had positive effects also on the psychological status of the patient. Despite a number of favorable clinical, psychological and economic effects, however, some concerns arose about patient compliance and nutritional status [134].

Learning from these experiences and in order to improve patient adherence, Caria et al. proposed a new combined Diet-Dialysis program [31]. Included in this program were patients with a higher residual renal function and the diet provided a less severe protein (0.6 g/kg/day) and phosphorus restriction on the non-dialysis days, but always respecting a high energy intake. A protein-free diet on the day of the dialysis session continued to be considered mandatory also in this program, in order to meet the increased nitrogen demand due to the losses of aminoacids during the dialysis session. When compared to a group of patients commencing at once a thrice-a-week hemodialysis schedule, patients on the combined Diet-Dialysis program showed a better preservation of both residual renal function and urine volume output, lower levels of β2-microglobulin, and better phosphate control. The use of erythropoietin stimulating agents, cinacalcet and non-calcium-non-aluminum phosphate binders were all significantly reduced in the experimental combined program. The hospitalization rate was also reduced. Half of the patients were still on Diet-Dialysis after 16 months [31]. An extension study showed a higher survival in patients who started such combined treatment as compared to those who commenced a regular thrice-a-week dialysis schedule.

The combined Diet-Dialysis approach could be considered a bridge therapy towards a more frequent hemodialysis, suited to selected and motivated patients. Under these controlled conditions, it can increase patient quality of life, prevent the rapid loss of either residual renal function or urine output volume and, moreover, it is cost-saving.

### Nutritional care management

#### Surveillance during low-protein diets in CKD

Two main issues set the stage for a successful LPD: prevention of protein-energy wasting and surveillance of adherence to dietary prescriptions. For this reason, patients on LPD require frequent monitoring by skilled personnel (nephrologists and dietitians). Nutritional monitoring should be based on an integrated evaluation approach, aimed at early diagnosis of PEW and identification of risk factors, in order to prevent any further depletion of protein and energy stores. Compliance evaluation requires active patient involvement and motivation. Literature data suggest that thoroughly monitored LPD in compliant patients, combined with adequate energy intake, are likely to be preventive rather than causative of PEW [64, 135–139].

In this clinical condition nutritional assessment should identify, characterize and stratify malnutrition, as well as monitor the response to therapy. However, early diagnosis of PEW risk is the key issue. No nutritional parameter taken individually can accomplish all these needs in renal patients. Currently, the available bedside methods, though not expensive, are not accurate and reproducible enough for the quantitative assessment of nutritional status. Moreover, they are quite often influenced by non-nutritional factors [140–147]. Nevertheless, changes over time in selected parameters may represent an important element of diagnostic utility.

A recent consensus published by the International Society of Renal Nutrition and Metabolism has suggested an integrated algorithm for regular (every three months) nutritional monitoring in CKD patients [148]. Screening includes 4 categories of readily utilizable criteria for the monitoring of nutritional status and clinical diagnosis of PEW: biochemical parameters, body mass, muscle mass and dietary intake (Table 2) [140]. A fifth additional category that includes nutritional scoring systems can be used as a complementary monitoring tool of nutritional status. PEW is considered to be present when at least one criterion in three out of four categories is present [140]. Not all of these nutritional variables have been introduced in daily clinical practice. Even though each of them has specific limitations, the most commonly used are represented by serum body mass index, weight change, serum albumin levels, and protein intake evaluation, since they are simple, noninvasive, widely available and inexpensive [140]. Nutritional scoring systems such as the Subjective Global Assessment (SGA) of nutritional status or the Malnutrition Inflammation Score (MIS) are widely used in the nephrology care setting because they integrate dietary history, careful physical examination, and nutritional evaluation [140].

**Table 2:** Criteria suggested for the nutritional monitoring of patients with CKD in conservative treatment. Most nutritional variables should be obtained every 3 months

Category	Nutritional variable	Additionally useful variables
Biochemical markers	Albumin < 3,8 g/dL Total cholesterol < 100 mg/dL	Transferrin, prealbumin Inflammatory markers: CRP, Total lymphocyte count or percentage
Body mass	BMI < 23 Kg/m^2^ Unintentional weight-loss > 5 % in 3 months or > 10 % in 6 months Reduced fat mass < 10 %	Bioelectrical Impedance Analysis
Muscle mass	Reduction of muscle mass by 5 % in 3 months or 10 % in 6 months Reduced AMA by 10 % as compared to the 50^th^ percentile of the reference population	DEXA (6 months interval) CT and/or MRI (6 months interval) Measurements of muscle strength and function (for example handgrip, 6 min walking test)
Nutritional intake	Unintentional DPI < 0.6 g/kg/day for at least 2 months Unintentional DEI < 25 kcal/kg/day for at least 2 months	Appetite assessment questionnaires Food frequency and dietary recall questionnaires Measuring energy expenditure by indirect calorimetry Protein catabolic rate (PCR)
Nutritional scoring system	Subjective Global Assessment (SGA) Malnutrition-inflammation Score (MIS)	

*AMA* arm muscle area, *BMI* body mass index, *CRP* C-reactive protein, *CT* computed tomography, *DEI* dietary energy intake, *DEXA* dual energy X-ray absorptiometry, *DPI* dietary protein intake, *MRI* magnetic resonance

A regular and careful evaluation of energy intake is mandatory. In this regard, dietary history assessment by a skilled renal dietitian is critical. The application of periodic surveys and food diaries allows early intervention, i.e., when the intake of calories is reduced but nutritional status assessment has not yet detected any alteration in nutritional variables [140]. Also, an early detection of loss of appetite or anorexia may represent the first index of a high risk condition for PEW. Protein intake in clinically and metabolically stable patients (i.e. in absence of acute comorbidities, such as infection and surgery) can be derived from the calculation of protein catabolic rate (PCR), based on a protein nitrogen appearance calculation by 24-h urinary nitrogen excretion and body weight value [149].

In parallel to regular nutritional status monitoring and dietary intake evaluation, patient motivation is to be reinforced, as is the case for other chronic diseases [150]. This can be attained by promoting active involvement of patients and their families, taking into account the different cultural and community issues. Namely, food and supplement access, economic and religious issues, health literacy etc. [151, 152]. In this regard, it is likely that an interactive approach based on simple tools describing permitted nutrients and the risk associated with some categories of food and its additives/preservatives, as well as the possibility of a more varied diet could significantly improve motivation and dietary adherence of CKD patients [153, 154].

#### Awareness and new comprehensive strategies to manage CKD

According to the World Health Organization, prevention of chronic diseases is of vital importance [155]. Many chronic diseases can be prevented since they are caused by modifiable factors such as improper diet, smoking or unsuitable lifestyles [156].

Chronic kidney disease is widely present in the world, affecting around 10 % of the general population. In Italy the prevalence of CKD is 6.3 % in the adult population [157]. The number of patients starting dialysis programs is constantly increasing and, therefore, during the course of CKD it is mandatory to make the utmost effort to postpone the initiation of dialysis as long as possible. The majority of the measures required for this purpose are organizational, aimed at reducing as much as possible the barriers that prevent effective treatment during CKD, whether they involve medication, diet or lifestyle. Delaying the initiation of dialysis treatment also means significant savings for the health system [158]. To be sure, it has been proven that the deferral of one to two years of dialysis results in savings of around 30–50,000 euros per patient [159]. Therefore it is crucial to administer suitable treatments, as well as to organize services able to offer the best treatment programs to CKD patients not in dialysis. It is not enough to solely provide knowledge of the remedies for the most severe risk factors, since even after very serious episodes of the disease, the correction of mistaken patient habits is very limited [160]. One of the causes of this behaviour is the patient’s difficulty in understanding what the physician is telling him, yet at the same time lacking the courage to ask for clarification. At times, it is the physician who does not wish to, or is unable to, explain more clearly and in more detail. As a result, the patient may not follow the recommendations, or may do so only in part [161, 162].

The belief emerging around the world is that only an active and aware patient will decide to follow both suggested advice and treatment, according to a “chronic care model” [163]. The Italian Society of Nephrology has developed a CKD program which leans in this direction [164]. The program describes in detail all the efforts and procedures which medical staff should make in order to inform, educate and support the patient in his/her goal towards understanding the disease and the therapy. A recent ministerial decree regarding the management of CKD contains chapters regarding therapeutic education as fundamental and essential issues in the treatment of a patient with kidney disease.

There is now a need to set up a program involving multidisciplinary and coordinated action among physicians, nursing staff, dietitians, physiotherapists and psychologists, to be preceded by periodical meetings in which the entire team acquires the necessary expertise and establishes coordination between the various members of the team. Informative materials are also required for the users (brochures, manuals, videos, cd’s, etc.). Since the majority of patients are elderly and many of them prefer television viewing over reading, educational videos could encourage and motivate patients to suitably follow the recommendations provided. It is also essential to define when the patient should be called for educational and training meetings, as well as which member(s) of the team should preside at each consultation, whether the physician together with the dietitian, or the physician with the nurse. When dietary information is provided, the caregiver who prepares the daily meals (next of kin, care-worker or other individual) must be present. The available materials must be viewed together with a medical professional. Generally the healthcare assistant or nurse has greater affinity with the patient, to whom the patient can refer in order to clarify any doubts which may have arisen during the patient-doctor consultation. Therefore, the patient will be motivated to clarify and gain better understanding of the program that is being illustrated. If videos are available, access can be granted to the patient by sharing via DVD, USB sticks or YouTube links.

A periodical internal audit to evaluate the results of the strategies used and, if necessary, to modify them, is fundamental in guaranteeing the success of the entire program.

## Summary

Italian nephrologists have a longstanding tradition in implementing low protein diets in the treatment of CKD patients, with the aim of alleviating uremic symptoms through reducing the toxins derived from excessive-intake of unnecessary protein [1]. Another aim was to improve the nutritional aspects of the CKD patients by providing adequate energy intake with protein of high biological value, eventually supplemented with essential aminoacids or their ketoanalogs. A not excluded aim was the possibility of slowing down the progression of CKD or at least to delay the need for dialysis. The current issue of the journal is a clear demonstration of this expertise. Moreover, the current issue is also a strong demonstration of the renewed interest of Italian nephrologists in revisiting this established field, with the aim of not only reducing the protein intake of CKD patients but also of implementing a wider and more correct nutritional therapy [8]. The major aspects of this novel approach were to pay more careful attention to energy intake and to the quality of proteins, achieved by the use of new protein-free foods of greatly improved quality, and also by paying attention to phosphate and sodium intakes. The low-protein diet program is now much more ambitious with the goal of improving the nutritional aspect of a population that is increasing in age, by carefully considering the amount and quality of protein intake, mainly the phosphate content and the amount of salt in the diet. The Idea was to reduce the progression of renal insufficiency through the reduction of proteinuria, through a better control of blood pressure values, and by the correction of metabolic acidosis. However, the experiences here reported clearly demonstrate the different dietary approaches in the various centres resulting from a well-recognised Italian flexibility and innovativeness in the field, both in treating non dialysis CKD patients, and in using low-protein diets as a bridge between conservative treatment and the start of chronic three-times a week dialysis therapy as an incremental dialysis approach. The main goal of this flexible approach is to favour patient compliance, which is a crucial factor in the successful implementation of a low-protein diet program.

## Abbreviations

ACEi, Angiotensin converting enzyme inhibitor; ARB, Angiotensin II receptor blocker; BW, Body weight; CKD, Chronic kidney disease; CKD-MBD, Chronic kidney disease mineral bone disease; CV,; Cardiovascular; DM, Diabetes mellitu; EAA, Essential aminoacids; EE,Energy expenditure; eGFR, Estimated glomerular filtration rate; ER, Energy requirement; ESRD, End stage renal disease; FGF-23, Fibroblast growth factor-23; GFR, Glomerular filtration rate; IGF-I, Insulin-like growth factor-I; KA, Ketoacids; LPD, Low-protein diet; PEW, Protein energy wasting; PI, Protein intake; RDA, Recommended dietary allowance; T1DM, Type-1 diabetes mellitus; T2DM, Type-2 diabetes mellitus; VLPD, Very low-protein diet

## References

[1] Giovannetti S, Maggiore Q (1964). A low nitrogen diet with proteins of high biological value for severe chronic uremia. Lancet.

[2] Brenner BM, Meyer TW, Hostetter TH (1982). Dietary protein intake and the progressive nature of kidney disease: the role of hemodynamically mediated glomerular injury in the pathogenesis of progressive glomerular sclerosis in aging, renal ablation, and intrinsic renal disease. N Engl J Med.

[3] Mitch WE, Remuzzi G (2004). Diet for patients with chronic kidney disease, still worth prescribing. J Am Soc Nephrol.

[4] Maschio G, Oldrizzi L, Tessitore N, D’Angelo A, Valvo E, Lupo A (1982). Effects of dietary protein and phosphorus restriction on the progression of early renal failure. Kidney Int.

[5] Barsotti G, Giannoni A, Morelli E, Lazzeri M, Vlamis I, Baldi R, Giovannetti S (1984). The decline of renal function slowed by very low phosphorus intake in chronic renal patients following a low nitrogen diet. Clin Nephrol.

[6] Isakova T, Wolf MS (2010). FGF23 or PTH: which comes first in CKD?. Kidney Int.

[7] Zoccali C, Ruggenenti P, Perna A, Leonardis D, Tripepi R, Tripepi G (2011). Phosphate may promote CKD progression and attenuate renoprotective effect of ACE inhibition. J Am Soc Nephrol.

[8] Locatelli F, Del Vecchio L (2014). Protein restriction: a revisited old strategy with new opportunities?. Nephrol Dial Transplant.

[9] Tom K, Young VR, Chapman T, Masud T, Akpele L, Maroni BJ (1995). Long-term adaptive responses to dietary protein restriction in chronic renal failure. Am J Physiol Endocrinol Metab.

[10] D’Alessandro C, Rossi A, Innocenti M, Ricchiuti G, Bozzoli L, Sbragia G (2013). Dietary protein restriction for renal patients: don’t forget protein-free foods. J Ren Nutr.

[11] Barsotti G, Morelli E, Cupisti A, Meola M, Dani L, Giovannetti S (1996). A low-nitrogen low-phosphorus vegan diet for patients with chronic renal failure. Nephron.

[12] Cupisti A, Kalantar-Zadeh K (2013). Management of Natural and Added Dietary Phosphorus Burden in Kidney Disease. Seminar Nephrology.

[13] Cupisti A, Morelli E, Meola M, Barsotti M, Barsotti G (2002). Vegetarian diet alternated with conventional low-protein diet for patients with chronic renal failure. J Ren Nutr.

[14] Aparicio M, Bellizzi V, Chauveau P, Cupisti A, Ecder T, Fouque D (2012). Ketoacid therapy in predialysis chronic kidney disease patients: final consensus. J Ren Nutr.

[15] Aparicio M, Bellizzi V, Chauveau P, Cupisti A, Ecder T, Fouque D (2013). Do Ketoanalogues Still Have a Role in Delaying Dialysis Initiation in CKD Predialysis Patients?. Semin Dial.

[16] Barsotti G, Ciardella F, Morelli E, Cupisti A, Mantovanelli A, Giovannetti S (1988). Nutritional treatment of renal failure in type 1 diabetic nephropathy. Clin Nephrol.

[17] Aparicio M, Gin H, Potaux L, Bouchet JL, Morel D, Aubertin J (1989). Effect of a ketoacid diet on glucose tolerance and tissue insulin sensitivity. Kidney Int.

[18] Gin H, Rigalleau V, Aparicio M (2000). Lipids, protein intake, and diabetic nephropathy. Diabetes Metab.

[19] Kaysen GA, Gambertoglio J, Jimenez I, Jones H, Hutchison FN (1986). Effect of dietary protein intake on albumin homeostasis in nephrotic patients. Kidney Int.

[20] Barsotti G, Morelli E, Cupisti A, Bertoncini P, Giovannetti S (1991). A special, supplemented “vegan” diet for nephrotic patients. Am J Nephrol.

[21] D’Amico G, Remuzzi G, Maschio G, Gentile MG, Gotti E, Oldrizzi L (1991). Effect of dietary proteins and lipids in patients with membranous nephropathy and nephrotic syndrome. Clin Nephrol.

[22] D’Amico G, Gentile MG, Manna G, Fellin G, Ciceri R, Cofano F (1992). Effect of vegetarian soy diet on hyperlipidaemia in nephrotic syndrome. Lancet.

[23] Cupisti A, Bottai A, Bellizzi V, Brunori G, Cianciaruso B, De Nicola L, et al. Characteristics of patients with chronic kidney disease referred to a nephrology outpatient clinic: results of Nefrodata study. G Ital Nefrol. 2015;32(2). 26005945

[24] De Nicola L, Minutolo R, Chiodini P, Zoccali C, Castellino P, Donadio C (2006). at al. TArget Blood Pressure LEvels in Chronic Kidney Disease (TABLE in CKD) Study Group. Global approach to cardiovascular risk in chronic kidney disease: reality and opportunities for intervention. Kidney Int.

[25] Bellizzi V. Low-protein diet or nutritional therapy in chronic kidney disease? Blood Purif. 2013;36(1):41–6. doi:10.1159/000350585. 10.1159/000350585 23735624

[26] Locatelli F, Alberti D, Graziani G, Buccianti G, Redaelli B, Giangrande A (1991). Prospective, randomised, multicentre trial of effect of protein restriction on progression of chronic renal insufficiency. Northern Italian Cooperative Study Group Lancet.

[27] Kasiske BL, Lakatua JD, Ma JZ, Louis TA (1998). A meta-analysis of the effects of dietary protein restriction on the rate of decline in renal function. Am J Kidney Dis.

[28] Fouque D, Laville M. Low protein diets for chronic kidney disease in non-diabetic adults. Cochrane Database Syst Rev. 2009;8(3):CD001892. doi: 10.1002/14651858.CD001892.pub3. 10.1002/14651858.CD001892.pub3 19588328

[29] Morelli E, Baldi R, Barsotti G, Ciardella F, Cupisti A, Dani L (1987). Combined therapy for selected chronic uremic patients: infrequent hemodialysis and nutritional management. Nephron.

[30] Locatelli F, Andrulli S, Pontoriero G, Di Filippo S, Bigi MC (1994). Supplemented Low-Protein Diet and Once Weekly Hemodialysis. Am J Kidney Dis.

[31] Caria S, Cupisti A, Sau G, Bolasco P (2014). The incremental treatment of ESRD: A low-protein diet combined with weekly hemodialysis may be beneficial for selected patients. BMC Nephrol.

[32] Duranton F, Cohen G, De Smet R, Rodriguez M, Jankowski J, Vanholder R (2012). Normal and pathologic concentrations of uremic toxins. J Am Soc Nephrol.

[33] Lisowska-Myjak B (2014). Uremic toxins and their effects on multiple organ systems. Nephron Clin Pract.

[34] Vanholder R. Uremic toxins. In: UpToDate. 2015. http://www.uptodate.com/contents/uremic-toxins.

[35] Garibotto G, Sofia A, Saffioti S, Bonanni A, Mannucci I, Verzola D (2010). Amino acid and protein metabolism in the human kidney and in patients with chronic kidney disease. Clin Nutr.

[36] Niwa T (2010). Indoxyl sulfate is a nephro-vascular toxin. J Ren Nutr.

[37] Ellis RJ, Small DM, Vesey DA. Indoxyl sulphate and kidney disease: causes, consequences and interventions. Nephrology. 2016;21(3):170–7. doi:10.1111/nep.12580. 10.1111/nep.12580 26239363

[38] Lin CJ, Chen HH, Pan CF, Chuang CK, Wang TJ, Sun FJ, Wu CJ (2011). p-Cresylsulfate and indoxyl sulfate level at different stages of chronic kidney disease. J Clin Lab Anal.

[39] Barreto FC, Barreto DV, Liabeuf S, Meert N, Glorieux G, Temmar M (2009). European Uremic Toxin Work Group (EUTox). Serum indoxyl sulfate is associated with vascular disease and mortality in chronic kidney disease patients. Clin J Am Soc Nephrol.

[40] Imanishi Y, Koyama H, Inaba M, Okuno S, Nishizawa Y, Morii H, Otani S (1996). Phosphorus intake regulates intestinal function and polyamine metabolism in uremia. Kidney Int.

[41] KDIGO (2009). clinical practice guideline for the diagnosis, evaluation, prevention, and treatment of chronic kidney disease-mineral and bone disorder (CKD–MBD). Kidney Int.

[42] Nadkarni GN, Uribarri J (2014). Phosphorus and the kidney: what is known and what is needed. Adv Nutr.

[43] Da J, Xie X, Wolf M (2015). Serum Phosphorus and Progression of CKD and Mortality: A Meta-analysis of Cohort Studies. Am J Kidney Dis.

[44] du Cailar G, Ribstein J, Mimran A (2002). Dietary sodium and target organ damage in essential hypertension. Am J Hypertens.

[45] Verhave JC, Hillege HL, Burgerhof JG, Janssen WM, Gansevoort RT, Navis GJ (2004). Sodium intake affects urinary albumin excretion especially in overweight subjects. J Intern Med.

[46] Swift PA, Markandu ND, Sagnella GA, He FJ, Macgregor GA (2005). Modest Salt Reduction Reduces Blood Pressure and Urine Protein Excretion in Black Hypertensives. A Randomized Control Trial Hypertension.

[47] He FJ, Marciniak M, Visagie E, Markandu ND, Anand V, Dalton RN, MacGregor GA (2009). Effect of modest salt reduction on blood pressure, urinary albumin, and pulse wave velocity in white, black, and Asian mild hypertensives. Hypertension.

[48] Dobre M, Rahman M, Hostetter TH (2015). Current status of bicarbonate in CKD. J Am Soc Nephrol.

[49] Saikumar JH, Kovesdy CP (2015). Bicarbonate Therapy in End-Stage Renal Disease: Current Practice Trends and Implications. Semin Dial.

[50] Driver TH, Shlipak MG, Katz R, Driver TH, Shlipak MG, Katz R (2014). Low serum bicarbonate and kidney function decline: the Multi-Ethnic Study of Atherosclerosis (MESA). Am J Kidney Dis.

[51] Motil KJ, Matthews DE, Bier DM, Burke JF, Munro HN, Young VR (1981). Whole-body leucine and lysine metabolism: response to dietary protein intake in young men. Am J Physiol.

[52] Young VR (1987). Kinetics of human acid metabolism: nutritional implications and some lessons. McCollum Award Lecture. Am J Clin Nutr.

[53] Workeneh BT, Mitch WE (2010). Review of muscle wasting associated with chronic kidney disease. Am J Clin Nutr.

[54] Garibotto G, Bonanni A, Verzola D (2012). Effect of kidney failure and hemodialysis on protein and aminoacid metabolism. Curr Opin Clin Nutr Metab Care.

[55] Kopple JD, Greene T, Chumlea WC (2000). Relationship between nutritional status and the glomerular filtration rate: results from the MDRD study. Kidney Int.

[56] Lim VS, Kopple JD (2000). Protein metabolism in patients with chronic renal failure: role of uraemia and dialysis. Kidney Int.

[57] Bailey JL, Zheng B, Hu Z, Price SR, Mitch WE (2006). Chronic kidney disease causes defects in signaling through the insulin receptor substrate/phosphatidylinositol 3-kinase/Akt pathway: implications for muscle atrophy. J Am Soc Nephrol.

[58] Sun DF, Chen Y, Rabkin R (2006). Work-induced changes in skeletal muscle IGF-I and myostatin gene expression in uraemia. Kidney Int.

[59] Zhang L, Wang XH, Wang H, Du J, Mitch WE (2010). Satellite cell dysfunction and impaired IGF-I signaling cause CKD-induced muscle atrophy. J Am Soc Nephrol.

[60] Garibotto G, Paoletti E, Fiorini F, Russo R, Robaudo C, Deferrari G, Tizianello A (1993). Peripheral metabolism of branched-chain ketoacids in patients with chronic renal failure. Miner Electrolyte Metab.

[61] Williams B, Hattersley J, Layward E, Walls J (1991). Metabolic acidosis and skeletal muscle adaptation to low protein diets in chronic uremia. Kidney Int.

[62] Goodship THJ, Mitch WE, Hoerr RA, Wagner DA, Steinman TI, Young VR (1990). Adaptation to low-protein diets in renal failure: leucine turnover and nitrogen balance. J Am Soc Nephrol.

[63] Bernhard J, Beaufrere B, Laville M, Fouque D (2001). Adaptive response to a low-protein diet in predialysis chronic renal failure patients. J Am Soc Nephrol.

[64] Masud T, Young VR, Chapman T, Maroni BJ (1994). Adaptive responses to very low protein diets: the first comparison of ketoacids to essential aminoacids. Kidney Int.

[65] Avesani C, Kamimura MA, Cuppari L (2011). Energy expenditure in chronic kidney disease patients. J Ren Nutr.

[66] Monteon FL, Laidlaw S, Shaib JK, Kopple JD (1986). Energy expenditure in patients with chronic renal failure. Kidney Int.

[67] Kopple JD, Monteon FJ (1986). Effect of energy intake on nitrogen metabolism in non-dialyzed patients with chronic renal failure. Kidney Int.

[68] Ikizler TA, Greene JH, Wingard RL, Parker RA, Hakim RM (1995). Spontaneous dietary protein intake during progression of chronic renal failure. J Am Soc Nephrol.

[69] Human Energy Requirements. Report of a joint FAO/WHO/UNU expert consultation. In: FAO Food and Nutrition Technical Report Series 1. 2001. http://www.fao.org/docrep/007/y5686e/y5686e00.htm

[70] Dietary Reference Intakes for Energy, Carbohydrate, Fiber, Fat, Fatty Acids, Cholesterol, Protein, and Amino Acids (Macronutrients). National Academic Press; 2005. 10.1016/s0002-8223(02)90346-9 12449285

[71] Rigalleau V, Combe C, Blanchetier V, Aubertin J, Aparicio M, Gin H (1997). Low protein diet in uremia: effects on glucose metabolism and energy production rate. Kidney Int.

[72] Protein and aminoacid requirements in human nutrition. Report of a Joint FAO/WHO/UNU Expert Consultation. In: WHO Technical Report Series 935. 2007. http://apps.who.int/iris/bitstream/10665/43411/1/WHO_TRS_935_eng.pdf.

[73] WHO. Sodium intake for adults and children - Guideline. 2012. http://apps.who.int/iris/bitstream/10665/77985/1/9789241504836_eng.pdf?ua=1&ua=1.

[74] O’Donnell MJ, Yusuf S, Mente A, Gao P, Mann JF, Teo K (2011). Urinary sodium and potassium excretion and risk of cardiovascular events. JAMA.

[75] Millward DJ (1999). Optimal intakes of protein in the human diet. Proc Nutr Soc.

[76] Cirillo M, Lombardi C, Chiricone D, De Santo NG, Zanchetti A, Bilancio G (2014). Protein intake and kidney function in the middle-age population: contrast between cross-sectional and longitudinal data. Nephrol Dial Transplant.

[77] Mozaffarian D, Fahimi S, Singh GM, Micha R, Khatibzadeh S (2014). Global Burden of Diseases Nutrition and Chronic Diseases Expert Group. Global sodium consumption and death from cardiovascular causes. N Engl J Med.

[78] Donfrancesco C, Ippolito R, Lo Noce C, Palmieri L, Iacone R, Russo O (2013). Excess dietary sodium and inadequate potassium intake in Italy: Results of the MINISAL study. Nutr Metab Cardiovasc Dis.

[79] KDIGO Guidelines, Chapter 3. Management of progression and complications of CKD. Kidney Int. 2013; Suppl 3:73–90. 10.1038/kisup.2012.66 PMC4283680 25598999

[80] De Nicola L, Minutolo R, Bellizzi V, Zoccali C, Cianciaruso B, Andreucci VE (2004). investigators of the TArget Blood Pressure LEvels in Chronic Kidney Disease (TABLE in CKD) Study Group. Achievement of target blood pressure levels in chronic kidney disease: a salty question?. Am J Kidney Dis.

[81] Bricker NS, Bourgoignie JJ, Weber H, Brenner BM, Rector FC (1976). The Kidney, Chapter 18. The renal response to progressive nephron loss.

[82] De Nicola L, Chiodini P, Zoccali C, Borrelli S, Cianciaruso B, Di Iorio B (2011). SIN-TABLE CKD Study Group. Prognosis of CKD patients receiving outpatient nephrology care in Italy. Clin J Am Soc Nephrol.

[83] Minutolo R, Locatelli F, Gallieni M, Bonofiglio R, Fuiano G, Oldrizzi L (2013). REport of COmorbidities in non-Dialysis Renal Disease Population in Italy (RECORD-IT) Study Group. Anemia management in non-dialysis chronic kidney disease (CKD) patients: a multicenter prospective study in renal clinics. Nephrol Dial Transplant.

[84] Vegter S, Perna A, Postma MJ, Navis G, Remuzzi G, Ruggenenti P (2012). Sodium intake, ACE inhibition, and progression to ESRD. J Am Soc Nephrol.

[85] Lambers Heerspink HJ, Holtkamp FA, Parving HH, Navis GJ, Lewis JB, Ritz E (2012). Moderation of dietary sodium potentiates the renal and cardiovascular protective effects of angiotensin receptor blockers. Kidney Int.

[86] D’Elia L, Rossi G, di CM S, Savino I, Galletti F, Strazzullo P (2015). Meta-Analysis of the Effect of Dietary Sodium Restriction with or without Concomitant Renin-Angiotensin-Aldosterone System-Inhibiting Treatment on Albuminuria. Clin J Am Soc Nephrol.

[87] D’Alessandro C, Piccoli GB, Calella P, Brunori G, Pasticci F, Bellizzi V, et al. “Dietaly”: practical issues for the nutritional management of CKD patients in Italy. BMC Nephrol. 2016; submitted. 10.1186/s12882-016-0296-5 PMC4966713 27473183

[88] Moranne O, Froissart M, Rossert J, Gauci C, Boffa JJ, Haymann JP (2009). NephroTest Study Group. Timing of onset of CKD-related metabolic complications. J Am Soc Nephrol.

[89] Bellizzi V, Scalfi L, Terracciano V, De Nicola L, Minutolo R, Marra M (2006). Early changes in bioelectrical estimates of body composition in chronic kidney disease. J Am Soc Nephrol.

[90] Cianciaruso B, Pota A, Pisani A, Torraca S, Annecchini R, Lombardi P (2008). Metabolic effects of two low protein diets in chronic kidney disease stage IV-V: a randomized controlled trial. Nephrol Dial Transpl.

[91] Milas NC, Nowalk MP, Akpele L, Castaldo L, Coyne T, Doroshenko L (1995). Factors associated with adherence to the dietary protein intervention in the Modification of Diet in Renal Disease Study. J Am Diet Assoc.

[92] Paes-Barreto JG, Silva MI, Qureshi AR, Bregman R, Cervante VF, Carrero JJ, Avesani CM (2013). Can renal nutrition education improve adherence to a low-protein diet in patients with stages 3 to 5 chronic kidney disease?. J Ren Nutr.

[93] Dolecek TA, Olson MB, Caggiula AW, Dwyer JT, Milas NC, Gillis BP (1995). Registered dietitian time requirements in the Modificaton of Diet in Renal Disease Study. J Am Diet Assoc.

[94] Bellizzi V, Di Iorio BR, Brunori G, De Nicola L, Minutolo R, Conte G (2010). Assessment of nutritional practice in Italian chronic kidney disease clinics: a questionnaire-based survey. J Ren Nutr.

[95] Pisani A, Riccio E, Bellizzi V, Caputo DL, Mozzillo G, Amato M (2015). 6-tips diet: a simplified dietary approach in patients with chronic renal disease.

[96] Cianciaruso B, Pota A, Bellizzi V, Di Giuseppe D, Di Micco L, Minutolo R (2009). Effect of a low- versus moderate-protein diet on progression of CKD: follow-up of a randomized controlled trial. Am J Kidney Dis.

[97] Kontessis P, Jones S, Dodds R, Trevisan R, Nosadini R, Fioretto P (1990). Renal, metabolic and hormonal responses to ingestion of animal and vegetable proteins. Kidney Int.

[98] Walser M, Coulter AW, Dighe S, Crantz FR (1973). The effect of ketoanalogues of essential aminoacids in severe chronic uremia. J Clin Invest.

[99] Marzocco S, Dal Piaz F, Di Micco L, Torraca S, Sirico ML, Tartaglia D (2013). Very low protein diet reduces indoxyl sulfate levels in chronic kidney disease. Blood Purif.

[100] Di Iorio BR, Minutolo R, De Nicola L, Bellizzi V, Catapano F, Iodice C (2003). Supplemented very low protein diet ameliorates responsiveness to erythropoietin in chronic renal failure. Kidney Int.

[101] Bellizzi V, Di Iorio BR, De Nicola L, Minutolo R, Zamboli P, Trucillo P (2007). ERIKA Study-group. Very low protein diet supplemented with ketoanalogs improves blood pressure control in chronic kidney disease. Kidney Int.

[102] Di Iorio B, Di Micco L, Torraca S, Sirico ML, Russo L, Pota A (2012). Acute effects of very-low-protein diet on FGF23 levels: a randomized study. Clin J Am Soc Nephrol.

[103] Di Iorio BR, Bellizzi V, Bellasi A, Torraca S, D’Arrigo G, Tripepi G, Zoccali C (2013). Phosphate attenuates the anti-proteinuric effect of very low-protein diet in CKD patients. Nephrol Dial Transplant.

[104] Goraya N, Simoni J, Jo C, Wesson DE (2012). Dietary acid reduction with fruits and vegetables or bicarbonate attenuates kidney injury in patients with a moderately reduced glomerular filtration rate due to hypertensive nephropathy. Kidney Int.

[105] Piccoli GB, Leone F, Attini R, Parisi S, Fassio F, Deagostini MC (2014). Association of low-protein supplemented diets with fetal growth in pregnant women with CKD. Clin J Am Soc Nephrol.

[106] Piccoli GB, Motta D, Martina G, Consiglio V, Gai M, Mezza E (2004). Low-protein vegetarian diet with alpha-chetoanalogues prior to pre-emptive pancreas-kidney transplantation. Rev Diabet Stud.

[107] Bellizzi V, Chiodini P, Cupisti A, Viola BF, Pezzotta M, De Nicola L (2015). Very low-protein diet plus ketoacids in chronic kidney disease and risk of death during end-stage renal disease: a historical cohort controlled study. Nephrol Dial Transplant.

[108] Willcox DC, Willcox BJ, Todoriki H, Suzuki M (2009). The Okinawan diet: health implications of a low-calorie, nutrient-dense, antioxidant-rich dietary pattern low in glycemic load. J Am Coll Nutr.

[109] Piccoli GB, Attini R, Vasario E, Gaglioti P, Piccoli E, Consiglio V (2011). Vegetarian supplemented low-protein diets. A safe option for pregnant CKD patients: report of 12 pregnancies in 11 patients. Nephrol Dial Transplant.

[110] Piccoli GB, Deagostini MC, Vigotti FN, Ferraresi M, Moro I, Consiglio V (2014). Which low-protein diet for which CKD patient? An observational, personalized approach. Nutrition.

[111] Piccoli GB, Ferraresi M, Deagostini MC, Vigotti FN, Consiglio V, Scognamiglio S (2013). Vegetarian low-protein diets supplemented with ketoanalogues: a niche for the few or an option for many?. Nephrol Dial Transplant.

[112] Piccoli GB et al. Diet as a System. BMC Nephrology 2016; *in press*10.1186/s12882-016-0413-5 PMC5142321 27927186

[113] AMD-SID. Standard italiani per la cura del diabete mellito. 2014. http://www.standarditaliani.it/skin/www.standarditaliani.it/pdf/STANDARD_2014_May28.pdf.

[114] Suckling RJ, He FJ, MacGregor GA. Altered dietary salt intake for preventing and treating diabetic kidney disease. Cochrane Database Syst Rev. 2010;8(12):CD006763. doi:10.1002/14651858.CD006763.pub2. 10.1002/14651858.CD006763.pub2 21154374

[115] Nezu U, Kamiyama H, Kondo Y, Sakuma M, Morimoto T, Ueda S. Effect of low-protein diet on kidney function in diabetic nephropathy: meta-analysis of randomized controlled trials. BMJ Open. 2013;3(5). doi:10.1136/bmjopen-2013-002934. 10.1136/bmjopen-2013-002934 PMC3664345 23793703

[116] Giordano M, Ciarambino T, Castellino P, Cataliotti A, Malatino L, Ferrara N (2014). Long-term effects of moderate protein diet on renal function and low-grade inflammation in older adults with type 2 diabetes and chronic kidney disease. Nutrition.

[117] Whitham D (2014). Nutrition for the prevention and treatment of chronic kidney Disease in Diabetes. Can J Diabetes.

[118] Pan Y, Guo LL, Jim HM (2008). Low-protein diet for diabetic nephropathy: a meta-analysis of randomized controlled trials. Am J Clin Nutr.

[119] Koya D, Haneda M, Inomata S, Suzuki Y, Suzuki D, Makino H (2009). Low- Protein Diet Study Group. Long-term effect of modification of dietary protein intake on the progression of diabetic nephropathy: a randomized controlled trial. Diabetologia.

[120] Barsotti G, Cupisti A, Barsotti M, Sposini S, Palmieri D, Meola M (1998). Dietary treatment of diabetic nephropathy with chronic renal failure. Nephrol Dial Transpl.

[121] Appel LJ, Moore TJ, Obarzanek E, Vollmer WM, Svetkey LP, Sacks FM (1997). A clinical trial of the effects of dietary patterns on blood pressure. DASH Collaborative Research Group. N Engl J Med.

[122] Vogt TM, Appel LJ, Obarzanek E, Moore TJ, Vollmer WM, Svetkey LP (1999). Dietary Approaches to Stop Hypertension: rationale, design, and methods. DASH Collaborative Research Group. J Am Diet Assoc.

[123] Feehally J, Baker F, Walls J (1988). Dietary protein manipulation in experimental nephrotic syndrome. Nephron.

[124] Ginevri F, Ghiggeri GM, Oleggini R, Barbano G, Bertelli R, Candiano G (1990). Low-protein diet and xanthine-metabolising enzymes in adriamycin nephrosis. Nephrol Dial Transplant.

[125] Peters H, Border WA, Noble NA (2000). Angiotensin II blockade and low-protein diet produce additive therapeutic effects in experimental glomerulonephritis. Kidney Int.

[126] Maroni BJ, Staffeld C, Young VR, Manatunga A, Tom K (1997). Mechanisms permitting nephrotic patients to achieve nitrogen equilibrium with a protein-restricted diet. J Clin Invest.

[127] Giordano M, De Feo P, Lucidi P, DePascale E, Giordano G, Cirillo D (2001). Effects of dietary protein restriction on fibrinogen and albumin metabolism in nephrotic patients. Kidney Int.

[128] Gansevoort RT, de Zeeuw D, de Jong PE (1995). Additive antiproteinuric effect of ACE inhibition and a low-protein diet in human renal disease. Nephrol Dial Transplant.

[129] Ciavarella A, Di Mizio G, Stefoni S, Borgnino LC, Vannini P (1987). Reduced albuminuria after dietary protein restriction in insulin-dependent diabetic patients with clinical nephropathy. Diabetes Care.

[130] Narita T, Koshimura J, Suzuki K, Murata M, Meguro H, Fujita H (1999). Effects of short-term glycemic control, low protein diet and administration of enalapril on renal hemodynamics and protein permselectivity in type 2 diabetic patients with microalbuminuria. Tohoku J Exp Med.

[131] Walser M, Hill S, Tomalis EA (1996). Treatment of nephrotic adults with a supplemented, very low-protein diet. Am J Kidney Dis.

[132] Chauveau P, Combe C, Rigalleau V, Vendrely B, Aparicio M (2007). Restricted protein diet is associated with decrease in proteinuria: consequences on the progression of renal failure. J Ren Nutr.

[133] Mitch WE, Sapir DG (1981). Evaluation of reduced dialysis frequency using nutritional therapy. Kidney Int.

[134] Locatelli F, Andrulli S, Pontoriero G, Di Filippo S, Bigi MC (1998). Integrated diet and dialysis programme. Nephrol Dial Transplant.

[135] Kopple JD, Coburn JW (1973). Metabolic studies of low protein diets in uremia. I. Nitrogen and potassium. Medicine (Baltimore).

[136] Kopple JD, Coburn JW (1973). Metabolic studies of low protein diets in uremia. II. Calcium, phosphorus and magnesium. Med (Baltimore).

[137] Kopple JD, Swendseid ME (1975). Evidence that histidine is an essential amino acid in normal and chronically uremic man. J Clin Invest.

[138] Lai S, Molfino A, Coppola B, De Leo S, Tommasi V, Galani A (2015). Effect of personalized dietary intervention on nutritional, metabolic and vascular indices in patients with chronic kidney disease. Eur Rev Med Pharmacol Sci.

[139] de Waal D, Heaslip E, Callas P. Medical nutrition therapy for chronic kidney disease improves biomarkers and slows time to dialysis. J Ren Nutr. 2016;26(1):1–9. doi:10.1053/j.jrn.2015.08.002. 10.1053/j.jrn.2015.08.002 26391566

[140] Fouque D, Kalantar-Zadeh K, Kopple J, Cano N, Chauveau P, Cuppari L (2008). A proposed nomenclature and diagnostic criteria for protein-energy wasting in acute and chronic kidney disease. Kidney Int.

[141] Obermayr RP, Temml C, Gutjahr G, Kainz A, Klauser-Braun R, Függer R, Oberbauer R (2009). Body mass index modifies the risk of cardiovascular death in proteinuric chronic kidney disease. Nephrol Dial Transplant.

[142] Cano M, Camousseigt J, Carrasco F, Rojas P, Inostroza J, Pardo A (2010). Body composition assessment in patients with chronic renal failure. Nutr Hosp.

[143] Pereira RA, Cordeiro AC, Avesani CM, Carrero JJ, Lindholm B, Amparo FC (2015). Sarcopenia in chronic kidney disease on conservative therapy: prevalence and association with mortality. Nephrol Dial Transplant.

[144] Macdonald JH, Marcora SM, Jibani M, Roberts G, Kumwenda MJ, Glover R (2006). Bioelectrical impedance can be used to predict muscle mass and hence improve estimation of glomerular filtration rate in non-diabetic patients with chronic kidney disease. Nephrol Dial Transplant.

[145] Erdogan E, Tutal E, Uyar ME, Bal Z, Demirci BG, Sayın B, Sezer S (2013). Reliability of bioelectrical impedance analysis in the evaluation of the nutritional status of hemodialysis patients - a comparison with Mini Nutritional Assessment. Transplant Proc.

[146] Avesani CM, Draibe SA, Kamimura MA, Cendoroglo M, Pedrosa A, Castro ML, Cuppari L (2004). Assessment of body composition by dual energy X-ray absorptiometry, skinfold thickness and creatinine kinetics in chronic kidney disease patients. Nephrol Dial Transplant.

[147] Bross R, Chandramohan G, Kovesdy CP, Oreopoulos A, Noori N, Golden S (2010). Comparing body composition assessment tests in long-term hemodialysis patients. Am J Kidney Dis.

[148] Ikizler TA, Cano NJ, Franch H, Fouque D, Himmelfarb J, Kalantar-Zadeh K (2013). International Society of Renal Nutrition and Metabolism. Prevention and treatment of protein energy wasting in chronic kidney disease patients: a consensus statement by the International Society of Renal Nutrition and Metabolism. Kidney Int.

[149] Clinical practice guidelines for nutrition in chronic renal failure (2000). K/DOQI, National Kidney Foundation. Am J Kidney Dis.

[150] Pagato SL, Appelhans BM (2013). A call for the end to the diet debates. JAMA.

[151] Upadhyay A, Earley A, Haynes SM, Uhlig K (2011). Systemic review: Blood pressure target in chronic kidney disease and proteinuria as an effect modifier. Ann Intern Med.

[152] Calderia D, Amaral T, David C, Sampaio C (2011). Educational strategies to reduce serum phosphorus in hyperphosphatemic patients with chronic kidney disease: A systematic review with meta-analysis. J Ren Nutr.

[153] KDIGO (2013). Clinical Practice Guideline for Evaluation and Management of Chronic Kidney Disease. Kidney Int.

[154] D’Alessandro C, Piccoli GB, Cupisti A (2015). The “phosphorus pyramid”: a visual tool for dietary phosphate management in dialysis and CKD patients. BMC Nephrol.

[155] Ward BW, Schiller JS, Goodman RA. Multiple chronic conditions among US adults: a 2012 update. Prev Chronic Dis. 2014;17;11:E62. doi:10.5888/pcd11.130389. 10.5888/pcd11.130389 PMC3992293 24742395

[156] Stuart-Shor E (2004). A public health action plan to prevent heart disease and stroke: the mandate for prevention across the continuum of care and across the lifespan. J Cardiovasc Nurs.

[157] De Nicola L, Donfrancesco C, Minutolo R, Lo Noce C, Palmieri L, De Curtis A (2015). ANMCO-SIN Research Group. Prevalence and cardiovascular risk profile of chronic kidney disease in Italy: results of the 2008–12 National Health Examination Survey. Nephrol Dial Transplant.

[158] Mennini FS, Russo S, Marcellusi A, Quintaliani G, Fouque D (2014). Economic effects of treatment of chronic kidney disease with low-protein diet. J Ren Nutr.

[159] Quintaliani G (2008). Socioeconomic aspects of dialysis treatment. G Ital Nefrol.

[160] Bowker TJ, Clayton TC, Ingham J, McLennan NR, Hobson HL, Pyke SD (1996). A British Cardiac Society survey of the potential for the secondary prevention of coronary disease: ASPIRE (Action on Secondary Prevention through Intervention to Reduce Events). Heart.

[161] WHO. Adherence to long-term Therapies: Evidence for action. 2003. http://www.who.int/chp/knowledge/publications/adherence_full_report.pdf?ua=1. 14562485

[162] Costa E, Giardini A, Savin M, Menditto E, Lehane E, Laosa O (2015). Interventional tools to improve medication adherence: review of literature. Patient Prefer Adher.

[163] Coleman K, Austin BT, Brach C, Wagner EH (2009). Evidence on the Chronic Care Model in the new millennium. Health Aff.

[164] Quintaliani G, Cappelli G, Lodetti L, Manno C, Petrucci V, Spinelli C (2009). Chronic kidney disease certification process manual by the Italian Society of Nephrology (SIN): Part I: clinical care delivery and performance measurements and improvement. J Nephrol.

